# An Evolving Definition of a “Healthy Diet”

**DOI:** 10.3390/nu15092212

**Published:** 2023-05-06

**Authors:** Arrigo F. G. Cicero, Federica Fogacci, Claudio Borghi

**Affiliations:** 1 Hypertension and Cardiovascular Risk Factors Research Unit, Medical and Surgical Sciences Department, Alma Mater Studiorum University of Bologna, 40100 Bologna, Italy; federica.fogacci@studio.unibo.it (F.F.); claudio.borghi@unibo.it (C.B.); 2 IRCCS AOU S. Orsola di Bologna, Via Massarenti 9, 40138 Bologna, Italy

Throughout life, most of us eat at least three meals a day for 365 days a year. It follows that, in a 90-year life, everyone is potentially exposed to food constituents (i.e., nutrients, bioactives, and other chemical compounds) more than 95 thousand times; therefore, dietary habits are a relevant determinant of health status.

In daily clinical practice, we usually discourage patients from eating foods deemed unhealthy, such as highly processed items. Even more rarely do we recommend that patients consume healthy foods, except as an alternative to junk foods. Nevertheless, it is well known that a healthy diet prevents several chronic and degenerative diseases and is the key to a healthy and long life [1]. However, what is a healthy diet and how do we define it? Recently, a large number of epidemiological studies have attempted to identify which dietary factors correlate with negative health outcomes, including death, loss of function, and lack of well-being. Determinants of positive health outcomes have been more rarely investigated, on the contrary.

In this Special Issue, Yeung et al. investigated dietary patterns that have been suggested to play a role in health in older age based on evidence from epidemiological data and clinical trials [2]. The authors concluded that dietary patterns focused on plant-based foods usually correlate with better aging outcomes; specific recommendations on this topic should also account for ethnicity, geographical location, and cooking methods as confounding factors. Then, dietary guidelines should be culturally adapted to achieve higher adherence and more favorable outcomes. Moreover, we should also acknowledge that dietary recommendations are usually based on observations from long-term population studies that allow us to conclude the types and qualities of food that were eaten 15–30 years before the guidelines’ release, and this is another critical issue.

Even if diet quality has been consistently associated with health outcomes, the energy distribution throughout the day is also able to exert a significant effect. A meta-analysis of seven large cohort studies (221,732 participants overall) has recently shown that skipping breakfast is associated with increased risk of cardiovascular disease (relative risk (RR): 1.22, 95% confidence interval (CI): 1.10–1.35) and all-cause mortality (RR: 1.25, 95% CI: 1.11–1.40) as compared with eating breakfast regularly [3]. A further meta-analysis including 399,550 individuals found a significant association between skipping breakfast and depression (pooled odd ratio (OR): 1.39, 95% CI: 1.34–1.44), stress (pooled OR: 1.23, 95% CI: 1.04–1.43), and psychological distress (pooled OR: 1.55, 95% CI: 1.47–1.62) [4]. In this Special Issue, Liu et al. highlighted the increased risk of suicide in adolescents enrolled in the Youth Risk Behavior Surveys (YRBSs), where being overweight/obese seems mediate the association between skipping breakfast and suicidality [5].

Of course, a number of foods can significantly affect human health beyond their nutritional value, and their positive (or negative) effects are mediated by specific bioactive compounds; an example is coffee. For decades, coffee has been thought to exert a negative impact on human health, causing insomnia, anxiety, gastritis, hypertension, and tachycardia. All these effects have been attributed to the coffee content in caffeine. However, the most recent literature suggests a positive effect of coffee intake (beyond the caffeine), that has been strongly associated with better outcomes in terms of all-cause mortality, cardiovascular disease mortality, and cancer-specific mortality [6]. In this Special Issue, Cicero et al. show that regular coffee drinking is associated with significantly lower levels of peripheral arterial pressure (both systolic blood pressure and pulse pressure) and central/aortic blood pressure than no coffee drinking [7]. Of course, this study has some limitations (e.g., individuals who had previously experienced caffeine side effects were excluded from the analysis), but it suggests that coffee consumption should not always be discouraged, as a moderate amount of regular coffee drinking could even protect against arterial aging.

Another issue that deserves to be addressed concerns the assessment of the impact of diet on human health. In effect, we usually study the impact of a single nutrient or different dietary patterns on hard outcomes (i.e., morbidity and mortality) or largely recognized risk factors (e.g., blood pressure, plasma concentrations of low-density lipoprotein cholesterol, and insulin resistance). However, the “-omics” assessment is the next frontier of epidemiologic research. In this Special Issue, Tanaka et al. evaluate the association of metabolomics biomarkers, different dietary patterns, and frailty in 806 community-dwelling older men and women from the Baltimore Longitudinal Study of Aging [8]. The authors identified multiple metabolite classes, further confirming the multiple effects of diet on health, and suggesting the “-omic” approach to diet requires more research, at least in geriatrics.

When we can more properly define what a “healthy diet” consists of (both from a qualitative and quantitative point of view), we will be able to implement nutritional advice at individual and population levels. In this context, improving digital healthy diet literacy is a new challenge and constitutes a new frontier in the communication of dietary education [9]. Furthermore, it must be recognized that people’s food choice is formed and constrained by circumstances that are essentially social and cultural and that dramatic events, such as the recent Coronavirus disease 19 (COVID-19) pandemic, strongly and quickly changed dietary habits at both individual and population levels [10]. Tailored dietary interventions and nutrition and lifestyle programs are more effective when repeated over time, as they help create habits.

In this context, epidemiological and clinical research aiming to finally define what a “healthy diet” means aims at creating more appropriate educational tools for the general population and specific algorithms to improve the management of patients affected by specific diseases, taking into account confounding factors such as sex, age, and ethnicity and considering that diet is a determinant of self-perceived quality of life and/or disease progression.
